# p75^NTR^ antagonists attenuate photoreceptor cell loss in murine models of retinitis pigmentosa

**DOI:** 10.1038/cddis.2017.306

**Published:** 2017-07-13

**Authors:** María Platón-Corchado, Pablo F Barcelona, Sean Jmaeff, Miguel Marchena, Alberto M Hernández-Pinto, Catalina Hernández-Sánchez, H Uri Saragovi, Enrique J de la Rosa

**Affiliations:** 1 Centro de Investigaciones Biológicas, CSIC, Madrid, Spain; 2 Lady Davis Institute-Jewish General Hospital, McGill University, Montreal, QC, Canada

## Abstract

ProNGF signaling through p75^NTR^ has been associated with neurodegenerative disorders. Retinitis pigmentosa (RP) comprises a group of inherited retinal dystrophies that causes progressive photoreceptor cell degeneration and death, at a rate dependent on the genetic mutation. There are more than 300 mutations causing RP, and this is a challenge to therapy. Our study was designed to explore a common mechanism for p75^NTR^ in the progression of RP, and assess its potential value as a therapeutic target. The proNGF/p75^NTR^ system is present in the dystrophic retina of the *rd10* RP mouse model. Compared with wild-type (WT) retina, the levels of unprocessed proNGF were increased in the *rd10* retina at early degenerative stages, before the peak of photoreceptor cell death. Conversely, processed NGF levels were similar in *rd10* and WT retinas. ProNGF remained elevated throughout the period of photoreceptor cell loss, correlating with increased expression of *α*_2_-macroglobulin, an inhibitor of proNGF processing. The neuroprotective effect of blocking p75^NTR^ was assessed in organotypic retinal cultures from *rd10* and *RhoP* mouse models. Retinal explants treated with p75^NTR^ antagonists showed significantly reduced photoreceptor cell death, as determined by the terminal deoxynucleotidyl transferase-mediated dUTP nick-end labeling (TUNEL) assay and by preservation of the thickness of the outer nuclear layer (ONL), where photoreceptor nuclei are located. This effect was accompanied by decreased retinal-reactive gliosis and reduced TNF*α* secretion. Use of p75^NTR^ antagonist THX-B (1,3-diisopropyl-1-[2-(1,3-dimethyl-2,6-dioxo-1,2,3,6-tetrahydro-purin-7-yl)-acetyl]-urea) *in vivo* in the *rd10* and *RhoP* mouse models, by a single intravitreal or subconjunctival injection, afforded neuroprotection to photoreceptor cells, with preservation of the ONL. This study demonstrates a role of the p75^NTR^/proNGF axis in the progression of RP, and validates these proteins as therapeutic targets in two different RP models, suggesting utility irrespective of etiology.

Retinitis pigmentosa (RP) refers to a group of inherited retinal dystrophies that are clinically similar despite arising from a large set of genetic mutations (http://www.sph.uth.tmc.edu/Retnet/disease.htm). These mutations usually trigger photoreceptor cell degeneration and death, leading to visual function decline and, eventually, blindness.^1^ While the time of onset and the rate of neurodegeneration are specified by the mutation, most, if not all, forms of RP share molecular and cellular mechanisms that include inflammation, microglial activation and reactive gliosis. These features are shared with other retinal diseases without a pure genetic origin, such as glaucoma, diabetic retinopathy and age-related macular degeneration.^2, 3^

Various therapeutic strategies for RP, including gene, cell and regenerative therapies, as well as pharmacological treatments, are gradually progressing from the animal models to clinical trials^4, 5, 6^ and http://clinicaltrials.gov/ct2/results?term=retinitis+pigmentosa. However, there is not yet any approved treatment for the neurodegenerative component of retinal diseases. The genetic complexity in the etiology of RP, comprising more than 300 described mutations in over 50 different genes, calls for the development of treatments targeting common mechanisms independently of the causative mutation. This would entail the detailed characterization of the processes leading to retinal deterioration, as a strategy to discover novel therapeutic targets.

As a monogenic genetic disease of high penetrance, a variety of animal genetic models recapitulate the signs and symptoms of human RP.^4^ A missense mutation in the *Pde6b* gene causes blindness in the *rd10* mouse model of autosomal recessive RP.^7^ The *RhoP* mouse model of autosomal dominant RP carries the mutant human rhodopsin Pro^347^Ser transgene.^8^ The course of the disease in these models recapitulate human progression and allows for effective experimental interventions.^9, 10, 11, 12, 13, 14, 15, 16^ We used these independent models of RP to study the involvement of the p75^NTR^/proNGF axis in the course of disease.

ProNGF is the precursor of mature NGF. ProNGF and NGF display opposite effects, depending on the receptor-complex stimulated. Both bind p75^NTR^ that mediates a neurotoxic effect. Indeed, p75^NTR^ activation is involved in several neurodegenerative conditions.^17, 18^ The deleterious signals of p75^NTR^ require an interacting protein sortilin, and are ligand dependent and activated by proNGF (although some signals can also be ligand independent).^18^ Conversely, NGF also binds the neuroprotective receptor Trk-A that can counterbalance p75^NTR^. Other proneurotrophins also bind p75^NTR^, although their functional impact is less characterized than in the case of proNGF.^18^

In retinal neurodegeneration associated with glaucoma and diabetic retinopathy, proNGF stimulates p75^NTR^-dependent production and secretion of TNF*α* and *α*_2_-macroglobulin (*α*2M) by activated glia.^19, 20, 21, 22, 23^ These are neurotoxic factors that trigger neuronal death in a paracrine manner. In addition, neurotoxic mechanisms are further promoted by *α*2M interacting with amyloid,^24^ and by *α*2M stabilizing proNGF and enhancing p75^NTR^ activation.^22^

Hence, expression of proNGF, TNF*α*, *α*2M, sortilin, as well as neuronal damage and glial activation, serve as markers of p75^NTR^-mediated neurodegeneration *in vivo*. Here, we characterize these components in the dystrophic *rd10* and *RhoP* retinas, as well as the use of p75^NTR^ antagonists to reduce neurotoxicity and delay neurodegeneration, both in retinal explants and *in vivo*. This work validates inhibition of p75^NTR^ as a therapeutic target for treatment of many forms of RP.

## Results

Previous findings demonstrate the involvement of the proNGF/p75^NTR^ system in optic nerve axotomy, diabetic retinopathy and glaucoma, diseases primarily affecting the retinal ganglion cell neurons.^19, 20^ We studied whether this mechanism is functional in a disease primarily affecting photoreceptor neurons, by quantifying the expression of p75^NTR^ and proNGF as well as the downstream effectors TNF*α* and *α*2M, and the glial activation during the neurodegenerative process associated with RP.

### Expression of p75^NTR^ in the *rd10* and the *RhoP* retina

**Figure 1:**
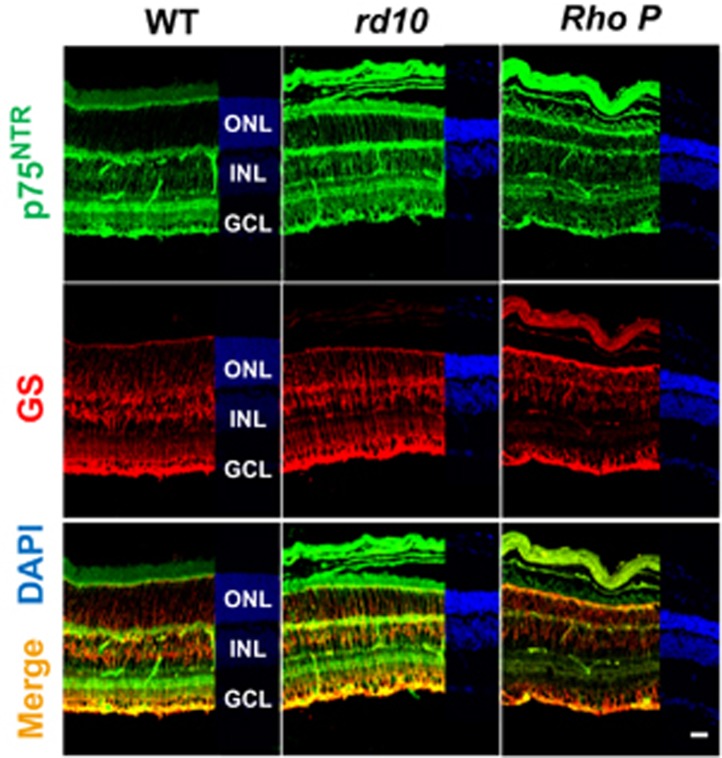
p75^NTR^ and GS immunostaining in the WT, *rd10* and *RhoP* retinas. Representative images of P21 retinal sections stained for p75^NTR^ (green) and GS (red). Nuclei are stained with DAPI. Scale bar: 20 *μ*m. ONL, outer nuclear layer; INL, inner nuclear layer; GCL, ganglion cell layer.

In both wild-type (WT) and the dystrophic *rd10* and *RhoP* retinas, p75^NTR^ immunostaining was mostly localized in Müller glia cells, as shown in P21 retinal sections (Figure 1). This observation was confirmed by costaining with the Müller glial cell marker glutamine synthetase (GS; Figure 1). Moreover, comparable p75^NTR^ immunostaining levels were found in both the WT and the dystrophic retinas.

### Unprocessed proNGF and inflammatory markers are increased in the *rd10* retina

**Figure 2:**
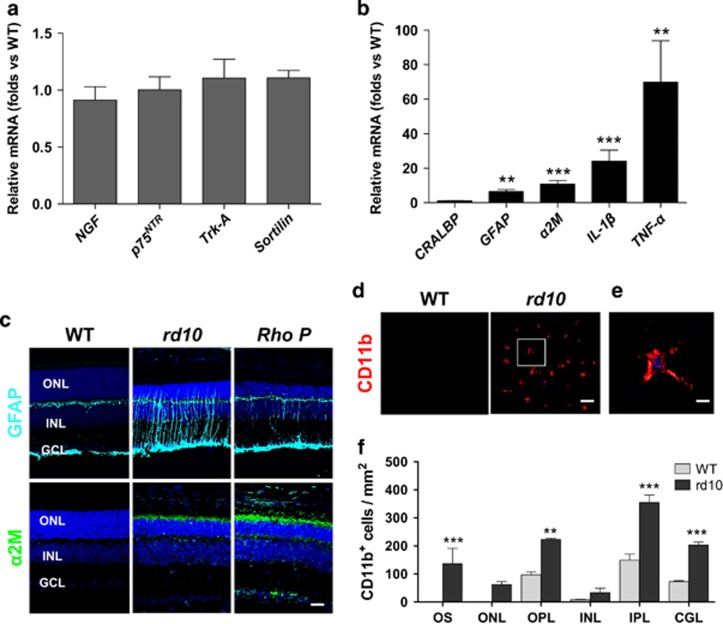
Expression of the NGF system components and inflammation markers. (**a**and **b**) RT-qPCR of WT and *rd10* retinas at P21. The levels of the different transcripts were normalized to the *TBP* RNA levels and relativized to WT levels (=1). (**c**) Representative images of P21 retinal sections from WT, *rd10* and *RhoP* mice, immunostained for glial fibrillary acidic protein (GFAP) (cyan) and *α*2M (green). Nuclei are stained with DAPI. (**d**) Representative confocal images at the level of the photoreceptor segments, in P23 WT and *rd10* retinas immunostained for CD11b. (**e**) Magnification of inset from the *rd10* retina. (**f**) Quantification of the number of CD11b-positive cells in the different layers of the WT (gray bars) and *rd10* (black bars) retinas. Bars represent the mean±S.E.M.; *n*≥3; **P<0.01, ***P<0.001. OS, outer segment; ONL, outer nuclear layer; ONL, outer nuclear layer; OPL, outer plexiform layer; INL, inner nuclear layer; IPL, inner plexiform layer; GCL, ganglion cell layer. Scale bars: (**c**) 30 *μ*m; (**d**) 50 *μ*m and (**e**) 20 *μ*m

We analyzed gene expression of the proNGF/p75^NTR^ system components in the retina of the *rd10* mouse, in comparison with WT, starting at stages previous to any observable sign of degeneration (P12) and during the initial period of photoreceptor cell degeneration and death, the time suited for a therapeutical treatment. Quantitative RT-PCR analysis did not show significant differences between WT and *rd10* retinas in the mRNA levels of either *NGF*, the *p75^NTR^* and *Trk-A* receptors, or the p75^NTR^ coreceptor *sortilin*, at P21, before the peak of photoreceptor cell death but when the degenerative process is well established (Figure 2a; WT expression level=1). Indeed, the mRNA profiles between P12 and P21 showed minor, no significant differences between WT and *rd10* retinas (Supplementary Figure 1). In contrast, the mRNA levels of the proinflammatory cytokines *IL-1β* and *TNFα*, as well as that of the neurodegeneration-associated, secreted protein, *α2M* were highly expressed at P21 in the *rd10* retina (Figure 2b; WT expression level=1). Moreover, a marked reactive gliosis in the *rd10* retina at this age was revealed by the increase of *GFAP* mRNA, whereas *CRALBP*, a general Müller glia cell transcript, remained unchanged with respect to WT retina (Figure 2b). *GFAP* and *α2M* overexpression was confirmed by immunolabeling of P21 retinal sections. The increased expression of GFAP and *α*2M was observed in both the *rd10* and the *RhoP* retinas (Figure 2c). GFAP immunoreactivity was associated to Müller gliosis, whereas *α*2M immunoreactivity was localized in the most external part of the dystrophic retinas, in close contact with the photoreceptor segments, retinal pigmented epithelium and choroids (Figure 2c and Supplementary Figure 2).

All these inflammatory signs concur with a marked increase of microglial cells in the *rd10* retina, determined at P23 (Figures 2d–f). Particularly relevant was the observed microglial infiltration in the surroundings of the degenerating *rd10* photoreceptor cells, including the outer plexiform layer, the ONL and the outer segments of photoreceptors, where *α*2M immunolabeling was localized.

**Figure 3:**
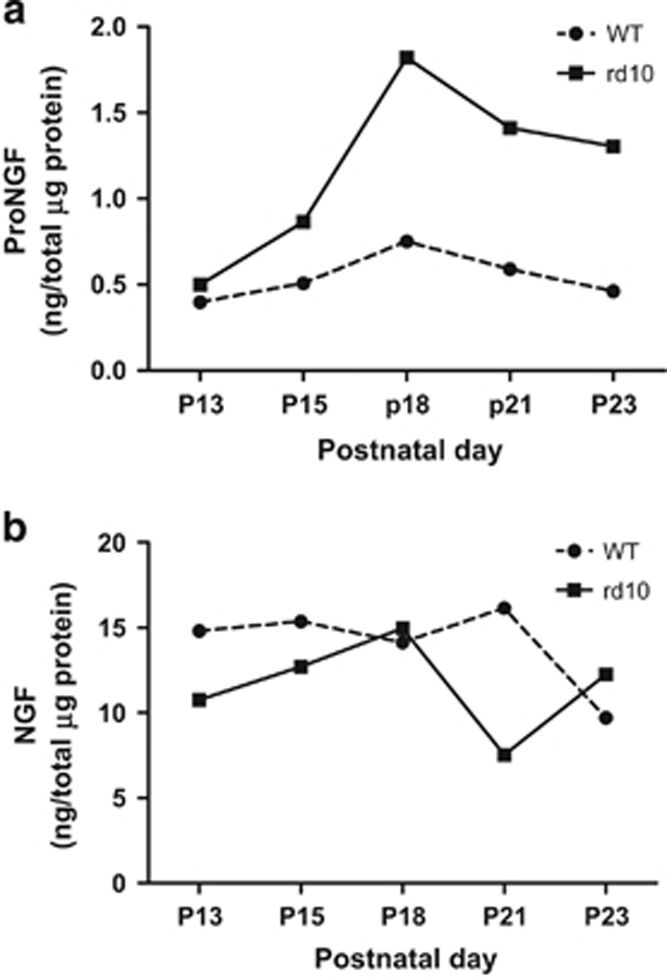
Precursor (proNGF) and processed protein (NGF) levels in the WT and *rd10* retinas. (**a**) ProNGF was quantified by enzyme-linked immunosorbent assay (ELISA) in four retina pools, at the indicated ages. (**b**) NGF was quantified by ELISA in four retina pools (the contralateral ones, from the same animals than proNGF) at the indicated ages. Two-way analysis of variance (ANOVA) for proNGF, F=12.99, *P*=0.0227; two-way ANOVA for NGF, F=1.49, *P*=0.2890

Next, we examined the persistence of unprocessed proNGF in the *rd10* retina (Figure 3). The levels of proNGF, as determined by ELISA, were similar in both the WT and the *rd10* retinas at P13, before any detectable photoreceptor degeneration. Strikingly, proNGF protein significantly increased in the *rd10* retina at P18, an early degenerative stage close to the beginning of photoreceptor cell death. ProNGF levels remained elevated in the *rd10* retina for the rest of the analyzed period, covering the interval of major photoreceptor cell loss, while proNGF in WT retinas persisted at similar, relatively low levels throughout the same time period (Figure 3a). Interestingly, a parallel determination of processed NGF did not show any increase in the *rd10* retina (Figure 3b).

These results suggest that elevated proNGF protein levels, likely activating p75^NTR^ in Müller glia cells, may have a role in the pathogenesis of RP.

### Blocking of p75^NTR^ decreases photoreceptor cell death and reactive gliosis in cultured *rd10* retinas

**Figure 4:**
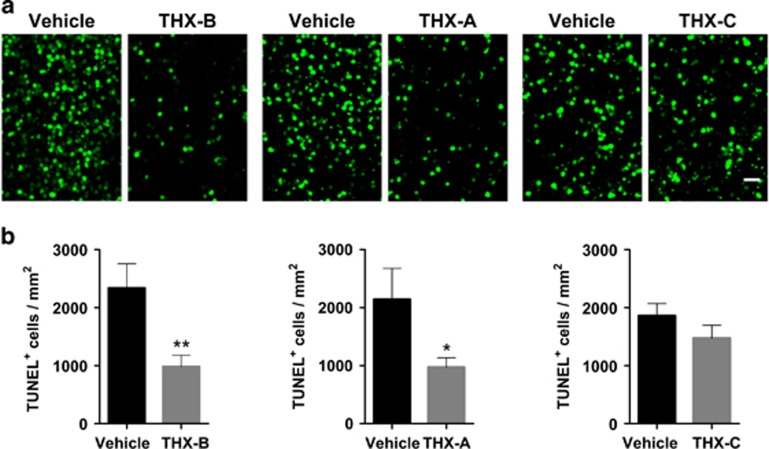
Effect of p75^NTR^ antagonists in *rd10* retinal explants. P22 *rd10* retinas were cultured for 24 h in the absence (vehicle) or presence of three different p75^NTR^ antagonists. (**a**) Representative images of retina explants stained for the TUNEL assay. (**b**) Quantification of the number of TUNEL-positive cells in the photoreceptor layer. Bars represent the mean±S.E.M.; *n*≥3; *P<0.05, **P<0.01. Scale bar: 20 *μ*m

We initially tested three small-molecule antagonists of p75^NTR 20^ in organotypic *rd10* retinal explants from P22 mice (Figure 4). Retinal explants recapitulate RP-associated photoreceptor cell death seen *in vivo* and allow for an easier primary screening before *in vivo* testing.^25^ In controls, very scarce photoreceptor cell death is observed in the time frame of the experiment in organotypic retinal explants from P22 WT mice (data not shown).

THX-B (1,3-diisopropyl-1-[2-(1,3-dimethyl-2,6-dioxo-1,2,3,6-tetrahydro-purin-7-yl)-acetyl]-urea) was very effective and reduced by 60% the density of TUNEL-positive nuclei in the photoreceptor layer of treated *rd10* retinas compared with vehicle-treated *rd10* retinas (Figure 4b). THX-A and THX-C (also known as LM11A-24^26^) are less active small-molecule analogs of THX-B, and exhibited a trend to reducing photoreceptor death. Another p75^NTR^ antagonist LM11A-31^26^ also partially reduced the density of TUNEL-positive nuclei in the retinal explants (Supplementary Figure 3a).

**Figure 5:**
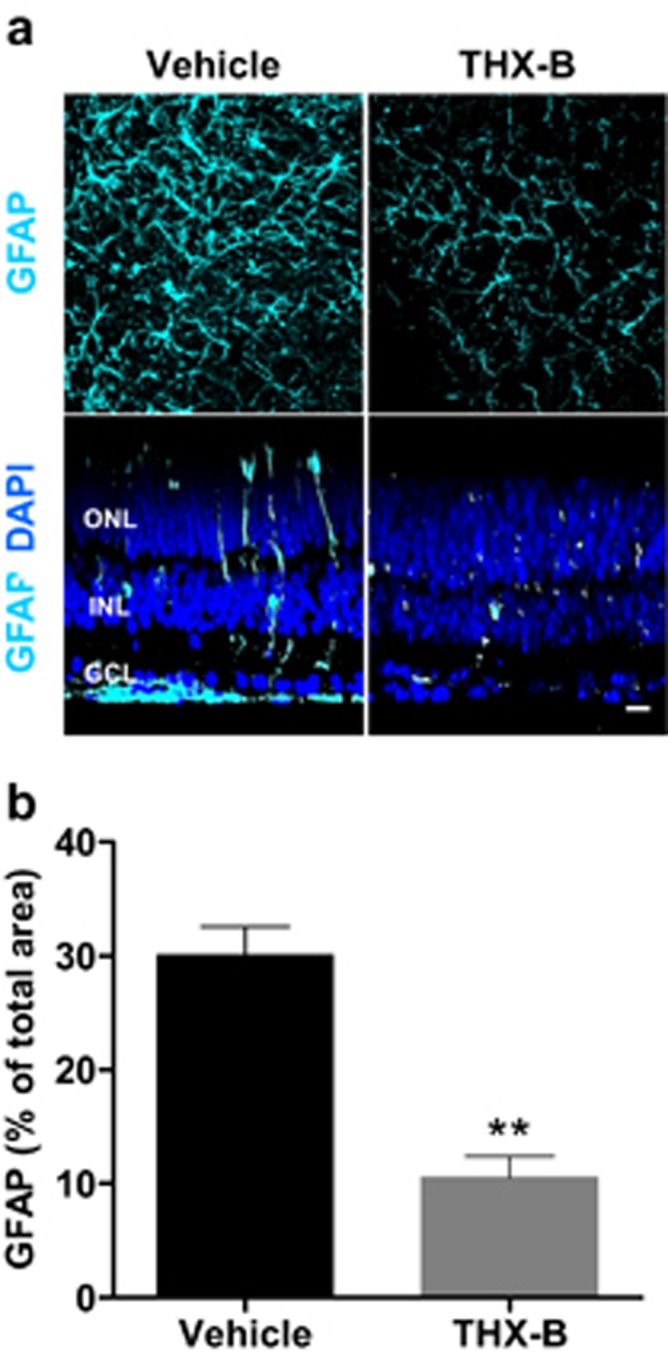
Effect of the p75^NTR^ antagonist THX-B on reactive gliosis in *rd10* retinal explants. P22 *rd10* retinas were cultured for 24 h in the absence (vehicle) or presence of THX-B. (**a**) Representative confocal optical sections of retinal whole mounts (xy, upper panels; xz, lower panels) stained for glial fibrillary acidic protein (GFAP) (cyan). Nuclei counterstained with DAPI (blue) are shown in the lower panels. (**b**) Quantification of the area filled with GFAP staining. Bars represent mean±S.E.M.; *n*≥3; **P<0.01. Scale bar: 20 *μ*m. ONL, outer nuclear layer; INL, inner nuclear layer; GCL, ganglion cell layer

In these experiments, THX-B had the highest relative potency in decreasing photoreceptor cell death; hence, THX-B was selected for a more detailed characterization of its effect *ex vivo* and *in vivo*. THX-B treatment of cultured P22 *rd10* retinas attenuated the thickening and enlargement of processes of astrocytes and Müller glia cells visualized by GFAP immunostaining (Figure 5).

### *In vivo* blocking of p75^NTR^ preserves the ONL

**Figure 6:**
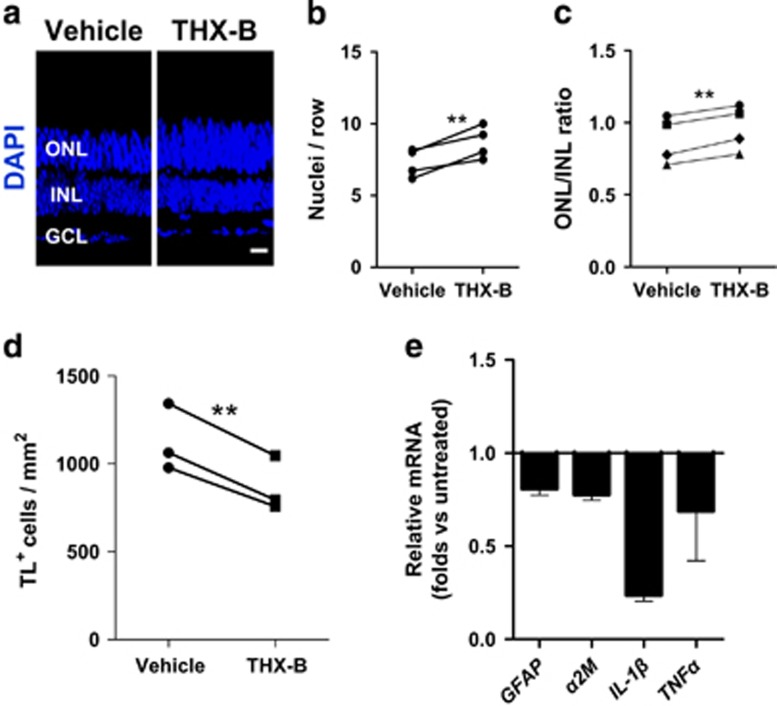
*In vivo* effect of the p75^NTR^ antagonist THX-B in the *rd10* retina. P17 *rd10* mice were intravitreally injected with THX-B in one eye and with vehicle in the contralateral eye, and analyzed at P22. (**a**) Representative sections. Nuclei are stained with DAPI. (**b**) Comparison of the photoreceptor number per row between the THX-B-treated eye and the control eye. Paired eyes are individually represented. (**c**) Comparison of the ONL/INL thickness ratio between the THX-B-treated eye and the control eye. Paired eyes are individually represented. (**d**) Quantification of the microglial (TL-positive) cell density in the THX-B treated retina *versus* the control retina from the same animal. Paired eyes are individually represented. (**e**) RT-qPCR of THX-B-treated and control retinas. The levels of the different transcripts were normalized to the *TBP* RNA levels and relativized to control retina levels. Bars represent mean±S.E.M.; *n*≥3; *P<0.05, **P<0.01; two-way analysis of variance (ANOVA) for RT-qPCR of treated *versus* control retinas, F=4.40, *P*=0.0467. Scale bar: 20 *μ*m. GCL, ganglion cell layer; INL, inner nuclear layer; ONL, outer nuclear layer; TL, tomato lectin

The results obtained in organotypic *rd10* retinal explants suggest that p75^NTR^ may be a pharmacological target for RP. Hence, we studied the *in vivo* effect of THX-B in the progression of the retinal degeneration in the murine RP models. A single intravitreal injection of THX-B at P17 in the *rd10* mouse eye elicited a neuroprotective effect on photoreceptor cells, observable at P22. The number of photoreceptor rows as well as the ONL/INL ratio were significantly higher in the treated eye than in the vehicle-injected eye from the same animal (Figures 6a–c). This concurred with a significant decrease in the total number of microglial cells in the treated retinas (Figure 6d), as well as a reduction in some of the inflammatory signs, such as the mRNA levels of *GFAP*, *α2M* and the proinflammatory cytokines *IL-1β* and *TNFα* (Figure 6e; untreated retinas expression level=1). Moreover, these effects concur with the reduced levels of TNF*α* secreted into the culture medium by LM11A-31-treated *rd10* retinal explants (Supplementary Figure 3c), supporting the proinflammatory role of p75^NTR^ in RP.

Taken together, our observations support the involvement of p75^NTR^ in the molecular and cellular processes occurring in the dystrophic retina and leading to degeneration.

**Figure 7:**
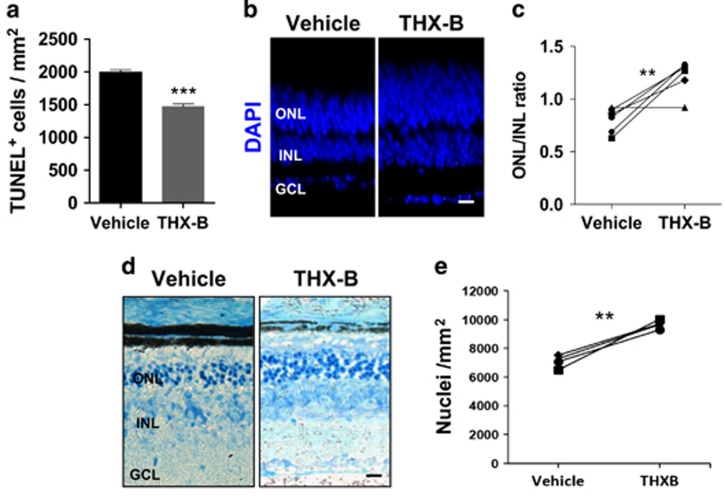
Effect of the P75^NTR^ antagonist THX-B, both *ex vivo* and *in vivo*, in the *RhoP* retina. (**a**) P18 *RhoP* retinas were cultured for 24 h in the absence (vehicle) or presence of the THX-B. Photoreceptor cell death was visualized by TUNEL and quantified. (**b**) P17 *RhoP* mice were intravitreally injected with THX-B in one eye and with vehicle in the contralateral eye, and analyzed at P22. Nuclei in whole-mount retinas were counterstained with DAPI. Representative cryosections. (**c**) Comparison of the ONL/INL thickness ratio between the THX-B-treated eye and the vehicle control eye. Paired eyes are individually represented. (**d**) P18 *RhoP* mice were subconjunctivally injected with THX-B in one eye and with vehicle in the contralateral eye and analyzed at P24. Representative cryosections stained with toluidine. (**e**) Comparison of the density of nuclei in the ONL between the THX-B-treated eye and the vehicle control eye. Paired eyes are individually represented. Bars represent the mean±S.E.M.; *n*≥3; **P<0.01; *** *P*<0.001. Scale bar: 20 *μ*m. GCL, ganglion cell layer; INL, inner nuclear layer; ONL, outer nuclear layer

To check the generality of our observations, we treated the *RhoP* mouse, which presents photoreceptor cell loss with a pace comparable to that of the *rd10* mouse,^8^ as well as similar features concerning, at least, p75^NTR^, GFAP and *α*2M expression patterns (Figures 1 and 2c). We first confirmed the effect of THX-B on photoreceptor cell death in P18 organotypic *RhoP* retinal explants. As in the case of the *rd10* retinas, THX-B significantly reduced the number of TUNEL-positive photoreceptor nuclei in comparison with untreated retinas (Figure 7a). We then tested the effect of THX-B *in vivo* in two different ways. A single THX-B intravitreal injection at P17 resulted, after 5 days, in higher ONL/INL thickness ratio than that of the contralateral vehicle-injected eye (Figures 7b and c), as previously shown for the *rd10* mouse (Figures 6a–c). Although intravitreal injection is a common clinical practice in human patients, we tested a less invasive delivery method. The *RhoP* mice were treated with a single subconjunctival injection of THX-B at P18 and the retinas were analyzed at P22. Again, the eyes treated with THX-B presented significant photoreceptor cell preservation as determined by the observed higher density of photoreceptor nuclei in the THX-B-treated eye than in the control eye (Figures 7d and e).

Altogether, our results confirm that p75^NTR^ has a relevant role in the RP-associated degenerative process, and constitutes a relevant therapeutic target to attenuate the course of this incurable group of inherited retinal dystrophies.

## Discussion

In the present work, we have analyzed the possible involvement of proNGF/p75^NTR^ signaling in the photoreceptor degeneration process associated with RP. Moreover, we have shown the potential therapeutic value of p75^NTR^ antagonists for RP treatment. THX-B administration reduced photoreceptor cell death *ex vivo* and *in vivo*, in two different RP models, thus providing an initial proof of concept on a possible therapy for RP. Remarkably, subconjunctival administration arises as a medical plausible treatment for RP, as well as for other retinal dystrophies.^27^

The proneurotophin–p75^NTR^ axis is emerging as a key player in the progression of several neurodegenerative pathologies.^18^ In the present study we have seen an increase in the proNGF protein levels in the *rd10* retinas before the massive loss of photoreceptors while no significant changes were observed in the processed NGF. Moreover, blocking p75^NTR^ decreased photoreceptor loss. Several studies in models of retinal and brain neurodegenerative damage showed a coordinated upregulation of several components of the proNGF-p75^NTR^ system.^28, 29^ In this study, we did not observe changes in the gene expression levels of *NGF*, *Trk-A*, *p75^NTR^* or its coreceptor *sortilin*. Indeed, the p75^NTR^ immunostaining pattern is similar in the WT, *rd10* and *RhoP* retinas, besides those associated with the degenerative changes. The nature of the damage as well as the age at which degeneration occurs may account for the differential regulation of the proNGF-p75^NTR^ system components in our model. In the *rd10* mouse, a model of early onset RP, the increase in proNGF at the protein level may be sufficient to trigger the neurotoxic response through p75^NTR^. In addition, p75^NTR^ may also bind other proneurotrophins^18^ (namely pro-BDNF, pro-NT3) in the degenerating *rd10* retinas, an alternative that will be addressed in future studies. The mechanistic role of the proNGF-p75^NTR^ signaling in neurodegenerative diseases has been widely attributed to its proapoptotic action. Indeed, p75^NTR^ belongs to the tumor necrosis factor superfamily characterized by the presence of an intracellular ‘death domain’ that interacts with components of the program cell death machinery.^30^ However, recent studies in retina have shown the contribution of proNGF-p75^NTR^ signaling to neuronal death through a non-cell-autonomous, paracrine mechanism. ProNGF acting on its p75^NTR^ receptor in Müller glial cells is able to induce TNF*α* production by Müller glial cells, which provokes ganglion cell degeneration.^23, 31^ We have observed a selective location of p75^NTR^ in Müller glial cells in WT, *rd10* and *RhoP* retinas (Figure 1) at the ages when RP-associated photoreceptor degeneration is taking place.

The source of proNGF may vary according to the type of injury and the affected tissue. It is worth recalling that microglial cells are a potential source of proNGF in the retina during development,^32, 33^ as well as in models of retinal dystrophies.^34^ In the *rd10* retina, microglia is highly activated and mobilized (Figures 2d–f), and may be the source of proNGF.

ProNGF is efficiently processed in the uninjured adult nervous system and very low levels of the proprotein form are detected under physiological conditions. The intracellular machinery that ensures the efficient conversion of proNGF into mature NGF is not well characterized. Retinal degeneration in the *rd10* mouse was accompanied by an increase of *α*2M, selectively located to the photoreceptor segment (Figures 2b and c and Supplementary Figure 2) and presumptively expressed by Müller glia cells,^35^ whose role in retinal neurodegeneration has been recently characterized in detail.^22, 24^ *α*2M binds to proNGF conferring resistance to processing and increased activity on p75^NTR^. The marked increase in the *α*2M in the *rd10* retina may account, at least in part, for the accumulation of proNGF. On the other hand, *α*2M binds to NGF and decreases its activity on the Trk-A receptor.

A hallmark of most forms of RP, despite their genetic origin, is the primary death of rod photoreceptors, in which the mutated gene exerts its function, followed by a secondary loss of cone photoreceptors.^5^ Photoreceptor degeneration is accompanied and followed by whole retinal remodeling, including retinal pigmented epithelium disorganization, inner neuronal connectivity alterations and vascular disorganization and regression. Concomitant to these alterations an inflammatory process takes place as shown by microglial recruitment, reactive gliosis of Müller glial cells and astrocytes, and a burst of proinflammatory cytokine expression.^3^ The hierarchies, respective roles and molecular action of microglia, Müller cells and astrocytes during *rd10* retinal degeneration remain to be elucidated. Interfering with one or several of these common players may provide a general therapy, despite the broad mutation spectrum of RP. Supporting this approach, suppression of microglial activation by minocycline treatment protected retinal structure and visual function in the *rd10* mouse.^14^ Further, TNF*α* inhibition by Adalimumab in the *rd10* mouse reduces photoreceptor cell death.^16^

In the present study, we have extended to RP the therapeutic potential of interfering with the proNGF/p75^NTR^ system described for other retinal dystrophies.^20, 21, 31^

## Materials and methods

### Animal procedures

The *rd10* mouse model of retinal degeneration is a homozygous recessive mutant for phosphodiesterase 6b (*Pde6b*^*rd10/rd10*^) on a C57BL/6 J background. The *RhoP* mouse model of retinal degeneration is a hemizygous dominant mutant that carries the mutant human *rhodopsin Pro^347^Ser* transgene on a C57BL/6 J background. WT mice of the same background were used as control. All animals were housed and handled in accordance with the ARVO statement for the Use of Animals in Ophthalmic and Vision Research, and European Union and Canadian guidelines. Mice were bred in the CIB and Lady Davis Institute core facilities.

### Intravitreal injections

*Rd10* and *RhoP* mice at P17 were anesthetized with isoflurane. Intravitreal injection was performed under an ophthalmoscope to visualize the retinal fundus. Using a Hamilton syringe (Hamilton AG, Bonaduz, Switzerland; Model 75 RN SYR) with a 33-gauge removable needle, the right eye was injected with 2 *μ*l of the p75^NTR^antagonist, THX-B (2 *μ*g/*μl* in 10% (v/v) DMSO in PBS), whereas the left eyes received 2 *μ*l of the vehicle. Animals were killed 5 days after injection, and eyes were processed for cryosectioning as described below. Both male and female mice were used for this study and at least three animals per group were used.

### Subconjunctival injection

*RhoP* at P18 were anesthetized with isoflurane. The conjunctiva was gently pulled from the sclera using tweezers. The conjunctiva of the right eye was injected with 30 *μ*l containing a total of 15 *μ*g THX-B (0.5 *μ*g/*μl* in 10% (v/v) DMSO in PBS). Half of the total volume of THX-B was delivered into the superior subconjunctival space and the other half into the nasal subconjunctival region using a microsyringe with a 33 G needle. The left eyes received 30 *μ*l of the vehicle. Animals were killed 4 days after injection and processed as above.

### p75^NTR^ antagonists synthesis

THX-B and the analogs have been described^20^ (US20100392647P patent by H Nedev and HU Saragovi). All compounds were purified by HPLC to >99%. The empirical formula of THX-B (F.W. 365) was confirmed by LC/MS, and the ^1^H NMR spectra (300 MHz) of compounds are consistent with the expected structures.

### Retina explant cultures

P20 and P22 *rd10* and P18 *RhoP* mice were killed, and their eyes were enucleated. Retinas were dissected and cultured free floating in M24 multiwell plates for 24 h in 1 ml DMEM/F12 medium containing N2 supplement, except insulin, and in the presence of the indicated p75^NTR^ antagonist THX-A, THX-B, THX-C, LM11A-31 (Tocris, Avonmouth, Bristol, UK), or vehicle. A dose range of 0.2–40 *μ*M drug concentrations were tested (only 20 *μ*M shown). Retinas were subsequently fixed in 4% (wt/vol) paraformaldehyde in phosphate buffer 0.1M, pH 7.4, for 1 h at RT and processed for detection of cell death and immunostaining.

### Cell death visualization and counting

Photoreceptor cell death was visualized by DNA fragmentation using the TUNEL assay (DeadEnd Fluorometric TUNEL System; Promega, Madison, WI, USA). Fixed whole-mount retinas were permeated with 2% (wt/vol) Triton X-100 (Merck, Darmstadt, Germany) in PBS for 2 h at RT, followed by incubation in 20 *μ*g/ml proteinase K (Merck) for 15 min at 37 °C and, subsequently, processed for TUNEL staining according to the manufacturer's instructions. After TUNEL labeling, the nuclei were counterstained with 0.05% (wt/vol) propidium iodide (Merck) in PBS, and the retinas were flat mounted in Fluoromount-G (Southern Biotechnology, Birmingham, AL, USA), and analyzed with a laser confocal microscope (TCS SP2; Leica Microsystems, Wetzlar, Germany). Serial optical sections were acquired with the × 63 objective every 1 *μ*m, covering the whole ONL thickness in four central fields around the optic nerve. Direct counting of TUNEL-positive cells was carried out using the FIJI open-source Software.^36^

### Immunolabeling of whole-mount retinas

Fixed whole-mount retinas were permeated with 2% (wt/vol) Triton X-100 in PBS, blocked in BGT (2.5% (wt/vol) BSA, 100 mM glycine, 0.25% (wt/vol) Triton X-100 in PBS) and incubated overnight at 4 °C, with the indicated primary antibodies or 2 h at RT with a biotinylated probe, after washing with TBS. Retinas were then washed with PBS and incubated for 2 h at RT with the secondary antibodies or streptavidin. The nuclei were counterstained with 4′,6-diamidino-2-phenylindole (DAPI) (Thermo Fisher Scientific, Waltham, MA USA), the retinas were mounted in Fluoromount-G and analyzed with a laser confocal microscope (TCS SP5 and TCS SP2; Leica Microsystems). Antibodies are listed below.

For determining microglia density, serial optical sections were acquired with a × 40 objective every 2.5 *μ*m through the whole retina in four central fields around the optic nerve. To measure the percentage of the area occupied by GFAP staining, serial optical sections were acquired with a × 40 objective every 2 *μ*m, covering the whole ganglion cell layer in 12 fields (four central, four medial and four peripheral) around the optic nerve. Confocal maxima of the ganglion cell layer Z sections were then converted into a black and white image and analyzed with the FIJI Software to calculate the percentage of total area filled with GFAP.

### Immunostaining of retinal sections

Animals were killed, their eyes were enucleated, fixed for 1 h in 4% (wt/vol) parafolmaldehyde in phosphate buffer 0.1 M, pH 7.4, and then cryoprotected by incubation in increasing concentrations of sucrose (15–50% in PBS). The eyes were then embedded in Tissue-Tek (Sakura, Leiden, The Netherlands), and frozen on dry ice. Cryostat sections (12 *μ*m) were mounted on Superfrost slides (Thermo Fisher Scientific) and dried at room temperature. For p75^NTR^ immunostaining, retinal sections were rehydrated in PBS, incubated with NaBH4 5 mg/ml in PBS for 5 min, followed by 0.1% (wt/vol) Triton X-100 and 5% (v/v) normal goat serum in PBS for 1 h. For immunostaining with the rest of the antibodies, retinal sections were permeated for 1 h with 0.2% (wt/vol) Triton X-100, and blocked with BGT for another hour. Following the incubation with the primary antibody overnight at 4 °C, the sections were washed with PBS and incubated for 1 h with secondary antibodies and DAPI. Then, sections were washed with PBS and coverslipped with Fluoromont-G. Antibodies are listed below.

To quantify the retinal structure using techniques unbiased to retinal location (i.e. the central regions of the retina are thicker than the peripheral regions), we compared the thickness of the ONL (containing primarily photoreceptors) and the corresponding INL (containing bipolar, horizontal and amacrine neuron as well as Müller glia cell bodies). Both ONL and INL were measured, and the ONL/INL ratios were calculated in treated eyes *versus* vehicle-injected contralateral eyes. Five sections per retina were analyzed. For each section, one photograph was taken for each of four defined retinal zones. In each photo three measurements of the ONL and INL thickness were performed in triplicate to obtain an average value per retinal zone per section. The measurements were carried out with the FIJI Software (using the ‘freehand line’ and ‘measure’ tools). Alternatively, the number of nucleus rows or the nuclear density in a determined retinal area were quantified in the same way to corroborate the neuroprotective effect and the accuracy of the ONL/INL ratio determination.


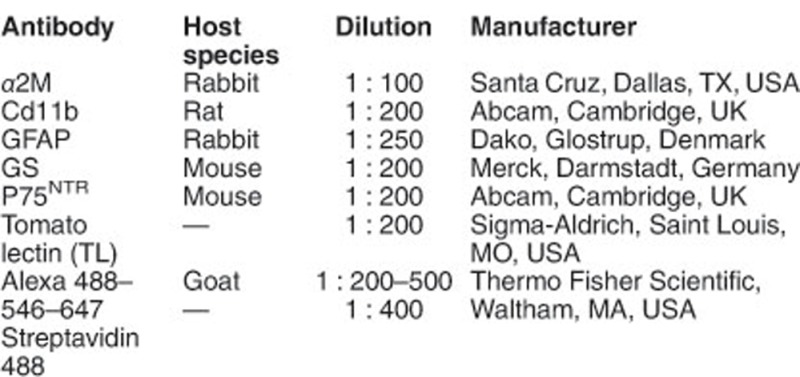


### ProNGF, NGF and TNF*α* levels determination

ProNGF and NGF levels were measured in retinal extracts from the same animals by ELISA assay (MyBioSource Inc., San Diego, CA, USA). For proNGF determination, four retinas per age were homogenized in PBS at 100 mg wet weight per ml and stored overnight at −20 °C. After two freeze–thaw cycles, the homogenates were centrifuged for 5000x*g* at 4 °C for 5 min. The supernatant was removed and assayed immediately following the manufacturer’s instructions. For NGF determination, the four contralateral retinas per age of the same animal used for proNGF determination were homogenized in PBS at 100 mg wet weight per ml and centrifuged for 1500x*g*for 15 min. The supernatant was removed and assayed immediately. TNF*α* concentration was measured in culture supernatant from retinal explants by ELISA assay (BioLegend, San Diego, CA, USA) following the manufacturer’s instructions.

### RNA isolation and quantitative PCR

Total RNA from individual retinas was extracted using the TRIzol reagent, and 2.5 *μ*g of RNA were typically reverse transcribed (RT) using the Superscript III Kit and random primers (all from Thermo Fisher Scientific). Quantitative PCR (qPCR) was performed with the ABI Prism 7900HT Sequence Detection System using TaqMan Universal PCR Master Mix, No-AmpErase UNG and the Taqman assays (listed below) for detection (all from Thermo Fisher).


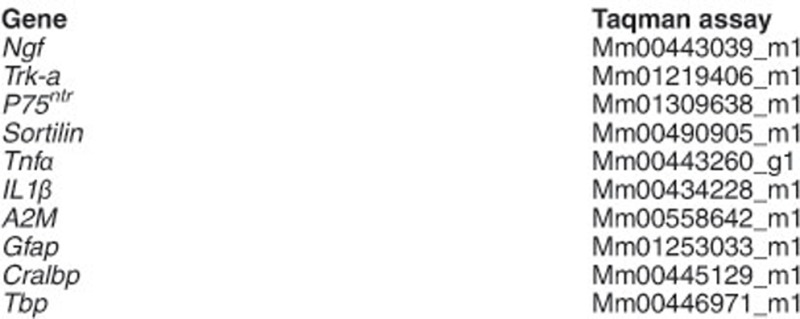


### Statistical analysis

Data size was estimated in accordance with previous literature, following the Reduction principle for animal research. At least three animals were analyzed per experimental point. Data points in graphs represent individual mice, and bars in all panels represent the mean and the standard error of the mean (S.E.M.).

Data were checked for normality using both D’Agostino–Pearson omnibus and Kolmogorov–Smirnov normality tests sequentially. Data were considered to fit a normal distribution only if they passed simultaneously both tests. For normal data, Fisher’s test was used to determine whether the variance of the samples analyzed was comparable (homoscedasticity). Normal data were compared using a two-way ANOVA with Bonferroni's multiple comparison test or an unpaired Student’s *T*-test, applying Welch’s correction in cases of non-homoscedasticity. In cases of non-normal samples, populations were compared using the Mann–Whitney nonparametric *U*-test. Outliers were detected by Grubbs’ outlier test, and excluded from further analysis. If previous literature allowed us to make a prediction about the result of the experiment, then one-sided test were applied. Otherwise, tests were two sided. All analyses were performed at a fixed 95% confidence interval, using the GraphPad Prism version 5.01 for Windows (GraphPad Software, San Diego, CA, USA; http://www.graphpad.com). Statistically significant differences are indicated as follows: *P<0.05; **P<0.01; ***P<0.001.
