# Supplementary material for: p75NTR antagonists attenuate photoreceptor cell loss in murine models of retinitis pigmentosa
Source: Cell Death Dis. 2017 Jul 13;8(7):e2922–. doi: 10.1038/cddis.2017.306 (PMC5550853; doi:10.1038/cddis.2017.306)
Supplement: Supplementary Figure 1 [file cddis2017306x1.pdf]

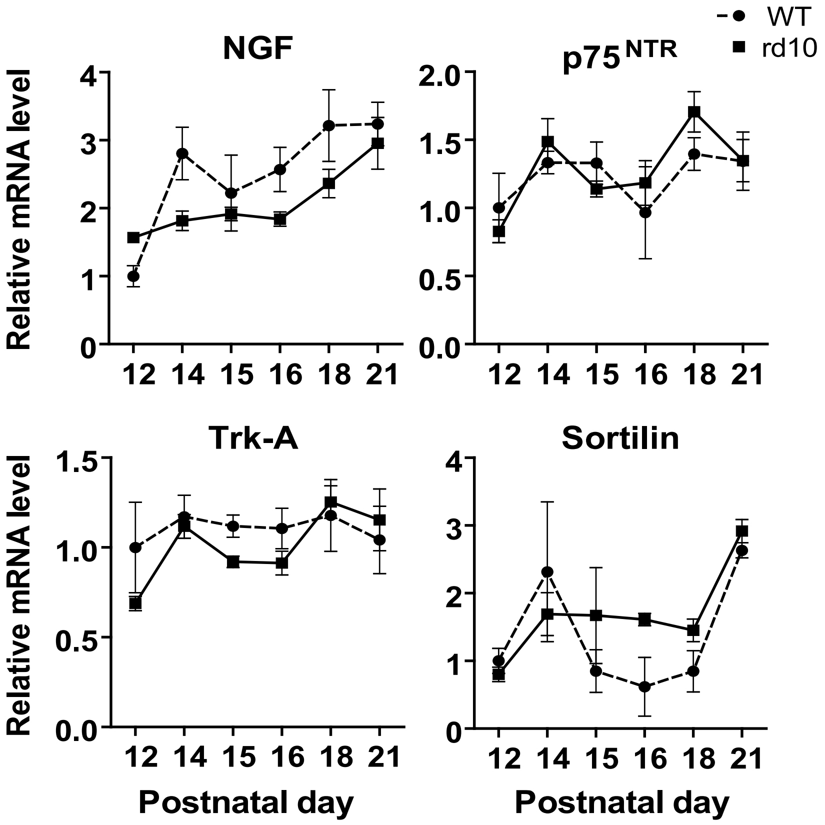

Supplementary Figure 1. NGF system components in the WT and *rd10* mouse retina. RT-qPCR of WT and *rd10* retinas at the indicated ages. The levels of the different transcripts were normalized to the TBP RNA levels and relativized to P12 WT level (= 1). Results represent the mean ± S.E.M. n≥3.
