# Supplementary material for: p75NTR antagonists attenuate photoreceptor cell loss in murine models of retinitis pigmentosa
Source: Cell Death Dis. 2017 Jul 13;8(7):e2922–. doi: 10.1038/cddis.2017.306 (PMC5550853; doi:10.1038/cddis.2017.306)
Supplement: Supplementary Figure 2 [file cddis2017306x2.pdf]

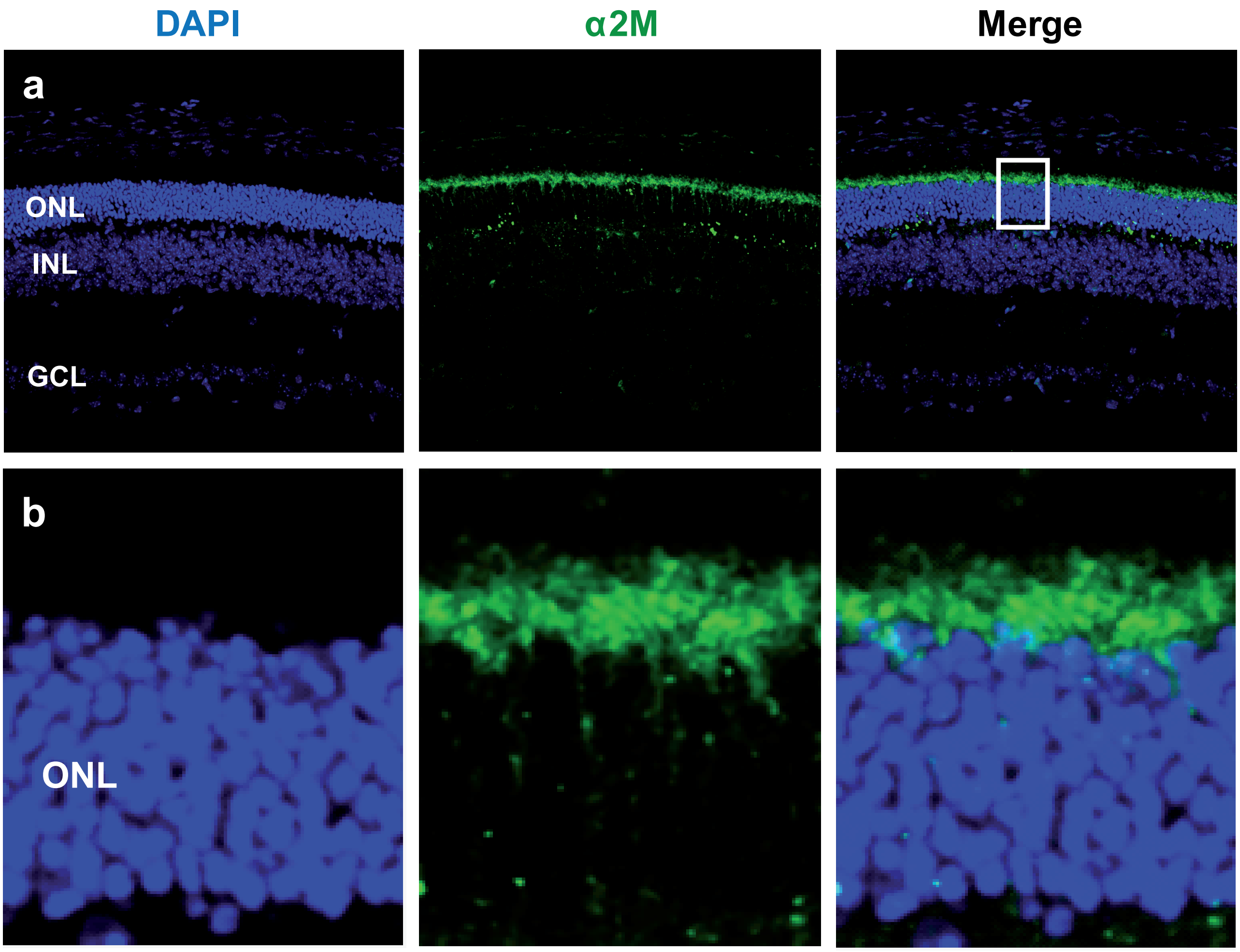

Supplementary Figure 2. α2M localization in the *rd10* retina. Magnification of α2M immunostaining (green) in a representative P21 retinal section from a *rd10* mouse, counterstained with DAPI (blue). (a) Section showed in Fig. 2c. (b) Magnification of the inset zone.
