# Supplementary material for: p75NTR antagonists attenuate photoreceptor cell loss in murine models of retinitis pigmentosa
Source: Cell Death Dis. 2017 Jul 13;8(7):e2922–. doi: 10.1038/cddis.2017.306 (PMC5550853; doi:10.1038/cddis.2017.306)
Supplement: Supplementary Figure 3 [file cddis2017306x3.pdf]

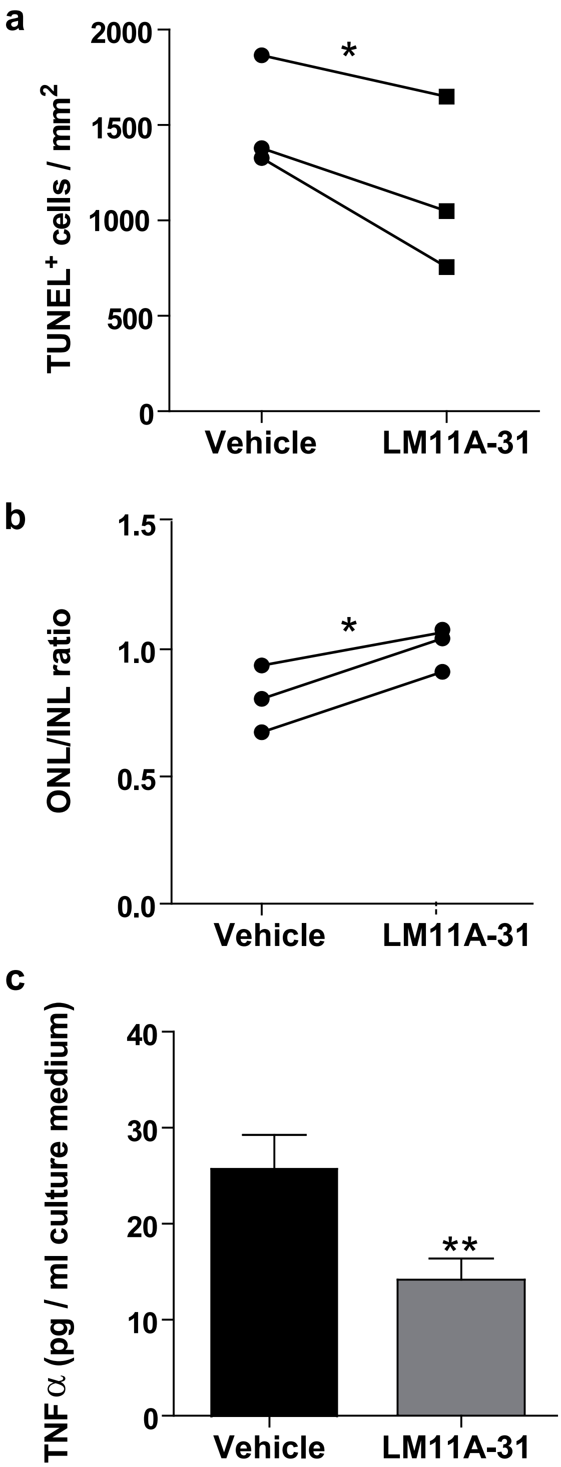

Supplementary Figure 3. Effect of $p75^{NTR}$ antagonist LM11A-31 in *rd10* retinal explants. Retinas were cultured for 24 h. Photoreceptor cell death (a) was quantified by TUNEL and the ONL/INL thickness ratio (b) was compared between paired treated and control eyes. TNFα concentration (c) was quantified in the culture media by ELISA. Bars represent mean ± S.E.M.; n=3 (a and b), n = 7 (c); *p < 0.05; **p < 0.01.
